# Selective Detection of an Infection Biomarker by an Osteo-Friend Scaffold: Development of a Multifunctional Artificial Bone Substitute

**DOI:** 10.3390/bios11120473

**Published:** 2021-11-24

**Authors:** Hye-In Kim, Naren Raja, Youngjun Choi, Jueun Kim, Aram Sung, Yeong-Jin Choi, Hui-suk Yun, Honghyun Park

**Affiliations:** 1 Department of Advanced Biomaterials Research, Ceramics Materials Division, Korea Institute of Materials Science (KIMS), 797 Changwon-daero, Seongsan-gu, Changwon 51508, Korea; hyeink@ucr.edu (H.-I.K.); naren1988@kims.re.kr (N.R.); dudwns028002@naver.com (Y.C.); hije112@kims.re.kr (J.K.); aramsung0@kims.re.kr (A.S.); jinchoi@kims.re.kr (Y.-J.C.); yuni@kims.re.kr (H.-s.Y.); 2 Department of Advanced Materials Engineering, Korea University of Science and Technology (UST), 217 Gajeong-ro, Yuseong-gu, Daejeon 34113, Korea

**Keywords:** CDHA, Au NPs, probe DNA, FRET, multifunctional artificial bone substitutes

## Abstract

Developments in three-dimensional (3D) printing technologies have led to many potential applications in various biomedical fields, especially artificial bone substitutes (ABSs). However, due to the characteristics of artificial materials, biocompatibility and infection remain issues. Here, multifunctional ABSs have been designed to overcome these issues by the inclusion of a biochemical modality that allows simultaneous detection of an infection biomarker by osteo-friend 3D scaffolds. The developed multifunctional scaffolds consist of calcium-deficient hydroxyapatite (CDHA), which has a similar geometric structure and chemical composition to human bone, and gold nanoparticles (Au NPs), which assists osteogenesis and modulates the fluorescence of labels in their microenvironment. The Au NPs were subsequently conjugated with fluorescent dye-labeled probe DNA, which allowed selective interaction with a specific target biomarker, and the fluorescent signal of the dye was temporally quenched by the Au NP-derived Förster resonance energy transfer (FRET). When the probe DNA unfolded to bind to the target biomarker, the fluorescence signal was recovered due to the increased distance between the dye and Au NPs. To demonstrate this sensing mechanism, a microbial oligonucleotide was selected as a target biomarker. Consequently, the multifunctional scaffold simultaneously facilitated osteogenic proliferation and the detection of the infection biomarker.

## 1. Introduction

Recent advances in three-dimensional (3D) printing technologies have led to the development of 3D scaffolds based on biopolymers, metals, and ceramic materials [1,2]. In terms of supply, mass production, and ethical issues, artificial 3D scaffolds have the capacity to overcome traditional limitations and have received attention as potential alternative devices [3,4]. They are currently fabricated from titania, polyurethane, and bioceramic materials that are biocompatible, mechanically strong, and have appropriate physical properties, thus facilitating modeling and modification. These diverse properties allow them to be widely applied as cardiovascular, skin, joint, and bone tissue substitutes, as well as personalized implants and clinical devices [2,5,6,7,8,9,10,11,12]. However, the disadvantages of artificial materials that are related to immunological rejection and infection remain issues to be overcome [13,14,15].

Currently, computed tomography (CT) scanning and magnetic resonance imaging (MRI) is performed to monitor the cellular environment of artificial tissue substitutes. However, these techniques are non-specific, making it difficult to distinguish types of microorganisms or cells in the microscopic environment. Subsequently, it is challenging to detect early stage infection or immune rejection. Furthermore, commonly used serological tests measure systemic inflammation markers, such as serum C-reactive protein levels, erythrocyte sedimentation rate, and blood plasma viscosity. This means it is impossible to determine the specific area of infection or make an early diagnosis. In cases of infection caused by an artificial device, often only when the device is surgically extracted can the cause of infection be determined using the pathoanatomical method. Hence, alternative types of devices are required to improve patient quality of life and eliminate the need for these non-specific methods, with their high cost and time inefficiencies. Ideally, the alternatives would be multifunctional devices and materials that meet structural and functional standards and simultaneously detect infection biomarkers.

Nanomaterials with controllable geometric structures and intrinsic optical properties have been widely applied in various biological fields, such as molecular diagnosis and tissue engineering [16,17]. In particular, gold nanoparticles (Au NPs) [18,19], silica nanoparticles [20], and quantum dots [21] have shown great potential in 3D printing in improving the functionality of 3D scaffolds by controlling or modulating osteogenic differentiation and bone regeneration, and detecting dental caries or periodontitis. These nanoparticle-assisted 3D scaffolds have been fabricated by mixing nanoparticles into a printing paste [20] or by conjugating the mature nanoparticles onto the scaffold surface [19]. Scaffolds produced using the former method have nanoparticles on both the surface and in the interior; therefore, a lot of the active nanoparticles are buried. Moreover, the nanoparticles within the paste easily settle down during the cementation step. In contrast, the latter method results in nanoparticles only immobilized on the scaffold surface. However, this requires additional steps, such as maturation of nanoparticles, purification, and chemical conjugation. To overcome these limitations and maximize the quality of active nanoparticles on the scaffold surface, a method is needed that immobilizes nanoparticles on the scaffold via an in situ production process.

In this study, a multifunctional artificial bone substitute was developed that is osteogenically active and simultaneously detects an infection biomarker. The scaffolds were produced via in situ growth of nanoparticles on osteo-friend 3D scaffolds. In a past study, Au NPs were grown in situ on calcium-deficient hydroxyapatite (CDHA) scaffolds, and enhanced osteogenesis was reported; however, their sensor-like potential has not yet been studied and reported [18]. To investigate the biomarker detection capability of the osteo-friend scaffolds, a target-specific oligonucleotide (probe DNA) was covalently conjugated to the Au NPs on the scaffolds, and the product was named Au-scaffold sensor. The probe DNA was labeled with an organic fluorescent dye; its emission range overlapped with the absorption of Au NPs. The developed Au-scaffold sensor has shown great potential for specific detection of an infection biomarker. In this case, the probe DNA targeted *Staphylococcus aureus*, and the scaffold retained its original osteogenic activity.

## 2. Materials and Methods

### 2.1. Fabrication of Ceramic Cement Scaffolds (CDHA)

A homogeneous mixture of CaCO_3_ (99.5%, Junsei Chemicals, Tokyo, Japan) and CaHPO_4_ (>98.5%, anhydrous, Sigma Aldrich, St. Louis, MO, USA) was prepared with a fixed molar ratio to obtain a final Ca/P of 1.5. The dried mixture was sintered at 1400 °C (heating rate = 5 °C/min) for 12 h, followed by quenching at room temperature [22]. The sintered powders were milled in a planetary ball mill at 250 rpm for 4 h in ethanol to obtain a uniform particle size. Using these powders, an extrudable alpha-Tricalcium phosphate (α-TCP) paste was prepared by mixing with a 1 wt% solution of hydroxypropyl methylcellulose (HPMC; 2600–5600 cP for 2% in H_2_O, Sigma Life Sciences; prepared in 30% ethanol). The powder to liquid ratio (P:L) was maintained at 1.67 for effective extrusion. The 3D scaffolds (8 mm diameter, 2 mm height) with uniform pore size were fabricated by layer-by-layer extrusion of ceramic pastes. Dried scaffolds were immersed in 1× phosphate-buffered saline (PBS) and incubated at 37 °C for 24 h. α-TCP transforms to CDHA by hydrolysis-based cement reaction. CDHA cement scaffolds were washed with deionized water three times and dried at 60 °C for further processing.

### 2.2. Functionalization of CDHA Scaffolds

**Figure 1:**
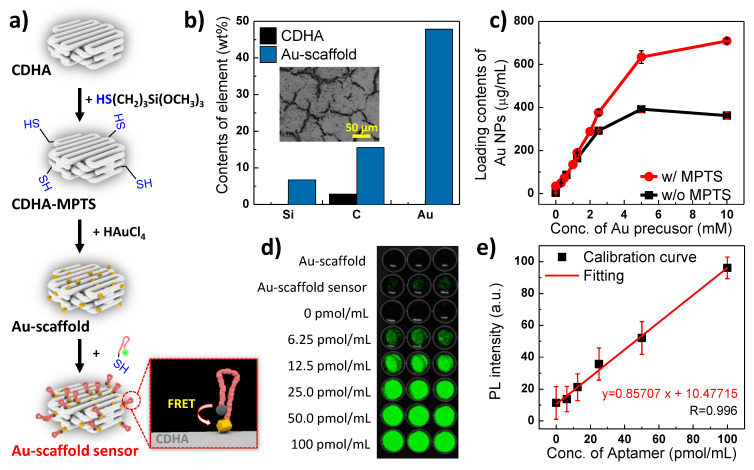
Synthesis and characterization of Au-scaffold sensor. (**a**) Schematic illustration of the synthesis of Au-scaffold sensor. (**b**) EDS analysis from the inset SEM image of Au-scaffold. (**c**) Quantitative analysis of Au NPs on the Au-scaffold by ICP analysis. (**d**) Fluorescence imaging for quantifying the amount of probe DNA on the Au-scaffold sensor. (**e**) Calibration curve for quantification of Probe DNA.

To introduce a thiol functional group to the surface of CDHA scaffolds, the mercaptopropyltrimethoxysilane (MPTS)-mediated silanization method was used, as shown in Figure 1a [23]. Briefly, a CDHA scaffold was immersed in 1 mL of absolute ethanol (99.5%, Sigma Aldrich, St. Louis, MO, USA). Then, 60 µL of 3-(trimethoxysilyl)propyl methacrylate and 10 µL of ammonium hydroxide solution (27.5%, Sigma Aldrich, St. Louis, MO, USA) were sequentially added to the CDHA scaffold mixture, and the reaction mixture was stirred using a thermomixer (13,000 rpm, 30 °C) to promote the silanization reaction. After incubating the reaction mixture for 12 h, the scaffold was washed with 10 mL of ethanol (70%) and distilled water (3rd distilled water, Sartorius, France) to remove the excess reactants and byproducts. The modified scaffold (denoted CDHA-MPTS) was air-dried. To grow the Au NPs on the CDHA-MPTS scaffold, 1 mL of gold precursor (10 mM HAuCl_4_, Sigma Aldrich, St. Louis, MO, USA) was added to the CDHA-MPTS and stirred for 12 h using a thermomixer (13,000 rpm, 30 °C). To remove the excess gold ions, the scaffold was rinsed 10 times with 1 mL of water each time. The resulting scaffolds were named Au-scaffolds.

### 2.3. Synthesis of Au-Scaffold Sensor

The *S. aureus* specific oligonucleotide (Bioneer or SFC probes, Korea) [24] was modified with a thiol group and fluorescein amidite (FAM) at the 3′ and 5′ end, respectively (5′-FAM-TGGACGTGGCTTAGCGTATATTT_15_-Thiol-3′). A disulfide bond between probe DNAs (4.26 nmol/mL) was cleaved using DL-dithiothreitol (DTT; 0.1 M in 0.18 M phosphate buffer, pH 8.0, Sigma Aldrich, St. Louis, MO, USA) for 1 h. The liberated probe DNAs were purified using an NAP-10 column (GE Healthcare, North Richland Hills, TX, USA) following the manufacturer’s instructions. Then, 0.7 mL of the cleaved probe DNA (0.5 nmol/mL) was mixed with a Au-scaffold in PB/SDS (0.01 M PB and 0.01% SDS) buffer solution for 1 h. For salification of the DNA, 2 M NaCl was added to the reaction mixture. The addition of the NaCl solution was repeated at 0.1 M NaCl increments until the concentration of NaCl was 1.0 M. The reaction mixture was stirred overnight. The desired Au-scaffold sensor was washed 10 times with 1 mL of PB/SDS buffer. Every mixing process was performed in UV-protected microtubes at 13,000 rpm (Thermomixer C, Eppendorf^®^, Germany).

## 3. Results and Discussion

### 3.1. Characterization of the Au-Scaffold Sensor

To characterize the modified CDHA scaffolds, elements of the Au-scaffold were compared with those of a pristine CDHA scaffold using Energy-Dispersive X-ray Spectroscopy (EDS) analysis of Scanning Electron Microscope (SEM) images (Figure 1b). Due to the silicon and carbon elements of the MPTS molecule, the Au-scaffold showed a higher content of Si and C than the CDHA scaffold. Au was observed only in the Au-scaffold. The thiol groups of CDHA-MPTS that supported the thiol-gold chemistry resulted in considerable immobilization of Au NPs on the scaffold surface [25]. The strong covalent bond of the thiol-Au interaction has reportedly higher binding affinity than the bond between Au and other functional groups, such as the amine functional group [26,27,28]. Therefore, the immobilized Au NPs on the Au-scaffold are more stable than other groups, such as amine or hydroxyl groups [18,19]. These strong interactions between the CDHA-MPTS thiol and the Au NPs resulted in Au NP loading of almost 709.0 µg/mL, while the pristine CDHA achieved 362.6 µg/mL under the same reaction conditions (Figure 1c). This controllable loading of Au NPs allowed the Au-scaffold to modulate probe DNA conjugation or Förster resonance energy transfer (FRET) efficiency. The effect of the Au NP quantity is examined in a later section.

The target-specific probe DNA was modified by the addition of FAM and a thiol functional group at the 5′ and 3′ ends, respectively. The probe DNA was immobilized onto the Au NPs of the Au-scaffold, and the product denoted Au-scaffold sensor. To quantify the immobilized probe DNAs, the probe DNAs were detached from the Au-scaffold sensor by treatment with 7.6 mL of DTT solution (25 mM) overnight. The supernatant containing the detached probe DNAs was collected, and fluorescence intensity was measured (Figure 1d). To clarify the absolute probe DNA concentration, the fluorescence intensity of a known concentration of probe DNA was measured under the same conditions in DTT solution (Figure 1d,e). The fluorescence intensity of the supernatants derived from the Au-scaffold sensor and Au-scaffold (w/o probe DNA, (-) control) were compared. The amount of probe DNA immobilized on a Au-scaffold sensor was calculated as 6.5 ± 15 nmol. Compared to the initial probe DNA quantity (7.2 nmol), the yield of immobilized probe DNA reached 90.3%. This highly efficient immobilization was achieved due to the thiol-Au interaction between the thiolate probe DNA and the Au-scaffold. Possible other interactions, such as hydrogen bonding, were investigated with Au-scaffold sensors treated with urea rather than DTT. Negligible fluorescence intensities were observed in the supernatants containing the detached probe DNA (data not supplied). Thus, the Au-scaffold was successfully synthesized using a thiol-Au interaction.

### 3.2. Optical properties of Au-Scaffold Sensor

**Figure 2:**
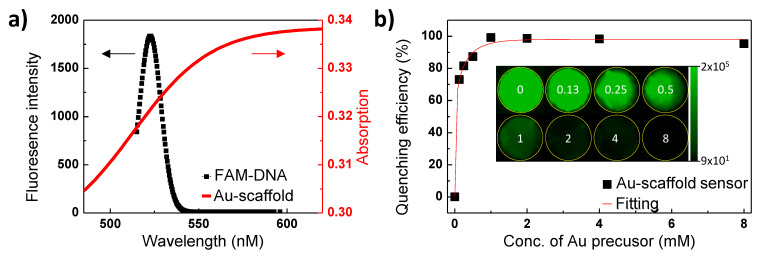
Optical properties of the Au-scaffold sensor. (**a**) The absorption of Au-scaffold (red line) and the fluorescence spectrum of FAM-labeled probe DNA (FAM-DNA). (**b**) FRET quenching efficiency of Au-scaffold.

The absorption of the Au-scaffold and the fluorescence of the FAM-labeled probe DNA were measured as shown in Figure 2a. The Au-scaffold exhibited a strong absorbance in the visible and near-infrared range. These strong and broad ranges of absorption indicate the presence of multiple Au NP structures on the Au-scaffold [29,30]. The absorption and fluorescence overlap between the Au-scaffold and FAM-labeled probe DNAs allows effective fluorescence quenching, as the Au-scaffold was used as a quencher in the FRET-based assays.

As the volume of Au NPs loaded onto the Au-scaffold sensor increased, the quenching efficiency exponentially increased (Figure 2b). The Au NP quantity on the Au-scaffold was controlled by the amount of Au precursor included in the fabrication step (0.13–8 mM). After conjugation of the FAM-labeled probe DNA, the fluorescence intensity of each Au-scaffold was measured (Figure 2b inset). As the amount of Au NPs on the Au-scaffold sensor increased, the fluorescence intensity exponentially decreased. Therefore, the FRET phenomenon was successfully observed with the Au-scaffold sensor. This approach, involving Au NPs and dye-labeled probe DNA, allows the 3D printed scaffold to be used as a sensor.

### 3.3. Optimization of Au-Scaffold Sensor

**Figure 3:**
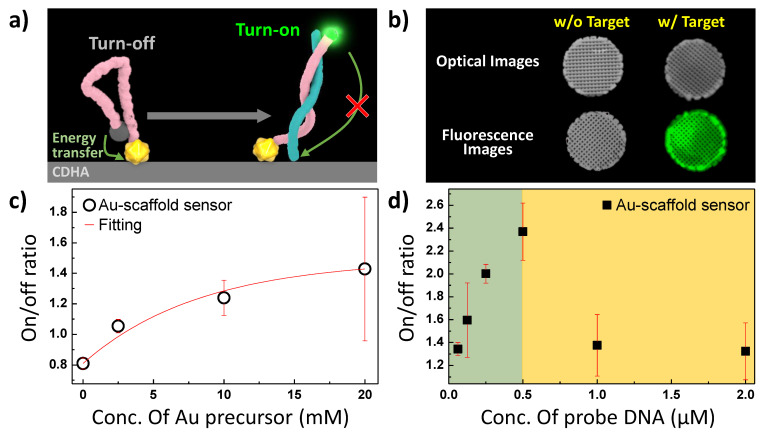
Optimization of the FRET system of Au-scaffold sensor. (**a**) Schematic illustration for the detection of target oligonucleotide on Au-scaffold sensor. (**b**) Fluorescence imaging of Au-scaffold sensor w/ and w/o of target treatment. (**c**) The ratio of PL turn on / turn off signal of Au-scaffold sensor as a function of Au NPs concentration and (**d**) FAM-labeled probe DNA.

The probe DNA structure on the Au-scaffold sensor was found to possess a folded structure in the free state, with a FRET distance of less than 10 nm (Figure 3a). When the probe DNA unfolded, the distance between the FAM and Au NPs reached approximately 13 nm. The FRET efficiency significantly decreases at distances greater than 10 nm [31]; therefore, the target binding event restored the fluorescence of FAM (Figure 3b). When the complementary DNA that was used as a model of the target was added to the Au-scaffold sensor, a fluorescence signal was emitted from all the tested Au-scaffold sensors. The amounts of quencher (Au NPs) and donor (FAM) were optimized to turn the signal on and off (Figure 3c,d). Increasing the amount of Au NPs reduced the signal, indicating that the turn on/off ratio could be manipulated.

While the concentration of the target oligonucleotide was kept constant (10 μM), the concentration of the Au precursor was increased from 2.5 mM to 20 mM (Figure 3c). As the concentration of the Au precursor increased, the fluorescence turn-on/off ratio increased. However, the reproducibility of the ratio of the Au-scaffold sensor with 20 mM Au precursor was unstable. The excess Au NPs were weakly immobilized on the Au-scaffold and could be detached from the scaffold during the assay process. Therefore, the optimal concentration of Au precursor for the Au-scaffold sensor was determined to be 10 mM.

The amount of FAM-labeled probe DNA was varied from 0.125 μM to 2.0 μM for the Au-scaffold sensor (Figure 3d). When the probe DNA concentration increased to 0.5 μM, the fluorescence turn on/off ratio increased due to the response signal of the dye increasing. However, the ratio suddenly decreased after 1.0 μM. The excess FAM of the probe DNA could not be quenched by the fixed amount of Au NPs; it allowed an increase in the turn-off intensity of the Au-scaffold sensor. From these results, the optimal concentration of FAM-labeled probe DNA was determined to be 0.5 μM.

### 3.4. Detection of Target Oligonucleotide by Au-Scaffold Sensor

**Figure 4:**
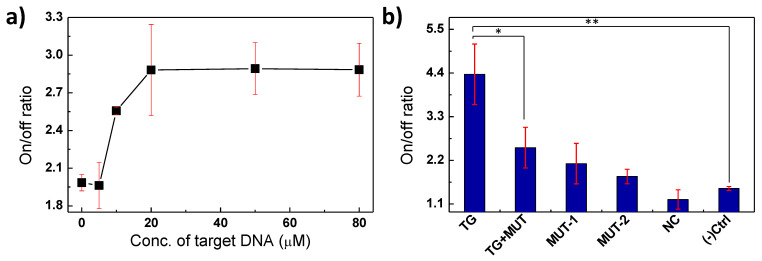
Detection of target oligonucleotide by Au-scaffold sensor. (**a**) Fluorescence turn-on/off ratio of Au-scaffold sensor as a function of the target oligonucleotide concentrations. (**b**) Fluorescence turn-on/off ratio of Au-scaffold sensor to target oligonucleotide (TG), target and mutation mixture (TG+MUT), a single (MUT-1) or double (MUT-2) mismatched mutation, or non-complementary DNA (NC). * and ** are indicated for *p* < 0.05 and *p* < 0.01, respectively.

The quenched fluorescence of the Au-scaffold sensor was gradually restored as the concentration of the target oligonucleotides increased, which showed that the fluorescence response was induced by the target oligonucleotide (Figure 4a). In addition, a non-complimentary DNA (NC, 5′-TCAGACAACCTGGCTTAGGGCACCTGCGTGGGAAACCTGC-3′) barely induced recovery of the quenched fluorescence of the Au-scaffold sensor (Figure 4b). In particular, single or double based-mismatched mutations (denoted MUT-1: 5′-AAT ATA CTC TAA GCC ACG TCC-3′ or MUT-2: 5′-AAT ATA CTC TAA GTC ACG TCC-3′, respectively) and a half mixture of target and mutations (denoted as TG+MUT) induced considerably smaller recoveries of the quenched fluorescence than the target sequence. These results indicate that the Au-scaffold sensor, with its FRET-based sensor, can selectively detect the specific target biomarker. Moreover, the probe DNA of the Au-scaffold sensor could be designed to target various biomolecules—not only oligonucleotides but also RNAs, peptides, proteins, and cells. Therefore, the developed Au-scaffold sensor could be applied to detect infection biomarkers with various structures.

### 3.5. Osteogenic Proliferation of the MG-63 Cell Line on the Au-Scaffold Sensor

**Figure 5:**
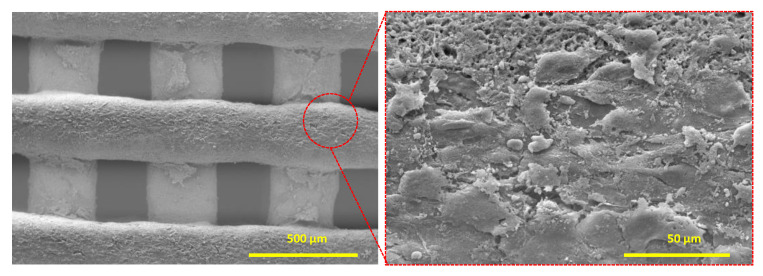
Surface structure of Au-scaffold sensor after incubation of MG-63 cells (10^6^ cells/ scaffold).

The synergetic effect of CDHA, a material commonly used in osteo-friend scaffolds, and Au NPs on the osteogenic proliferation of human mesenchymal stem cells is thought to assist osteogenesis of MG-63 cells [32,33,34,35]. Prolific cell growth on the Au-scaffold sensor was observed, and the cell attachment was confirmed when SEM images were analyzed (Figure 5). The overgrowth of MG-63 cells and their dendritic structure were clearly observed on the Au-scaffold sensor. This result indicates that the developed Au-scaffold sensor could be successfully used as a multifunctional artificial bone substitute.

## 4. Conclusions

An Au-scaffold sensor was developed as a multifunctional artificial bone substitute that was osteogenically active and simultaneously detected an infection biomarker. The scaffold was constructed by adding nanoparticles in situ to osteo-friend 3D scaffolds. Specifically, the developed Au-scaffold sensors consisted of in situ grown Au NPs on CDHA scaffolds and subsequently conjugated target-specific probe DNAs. The scaffolds facilitated the osteogenesis of MG-63 cells and detected specific target molecules. The developed Au-scaffold sensor has been shown to be a multifunctional artificial bone substitute and has great potential for specific detection of infection biomarkers. For further applications of the Au-scaffold sensor in vivo, it is required to investigate essential properties such as stability of probe DNA, tissue penetration of the imaging signals, and biocompatibility. Additionally, further in vivo study with infectious models is highly required to apply the developed Au-scaffold sensor to real applications, such as hematogenous implant infection models or bacteria sealed implant infection models.

## Data Availability

Not applicable.
